# The Relation of Lifestyle with Inflammation at the Time of Diagnosis in Patients with Colorectal Cancer

**DOI:** 10.3390/cancers15174307

**Published:** 2023-08-28

**Authors:** Anke H. C. Gielen, Jarno Melenhorst, Stephanie O. Breukink, Matty P. Weijenberg, Martijn J. L. Bours

**Affiliations:** 1 Department of Surgery, Maastricht University Medical Centre, 6229 HX Maastricht, The Netherlands; 2 School of Nutrition and Translational Research in Metabolism (NUTRIM), Maastricht University, 6229 ER Maastricht, The Netherlands; 3 GROW School for Oncology and Reproduction, 6229 ER Maastricht, The Netherlands; 4 Department of Epidemiology, Maastricht University, 6229 ER Maastricht, The Netherlands

**Keywords:** colorectal cancer, inflammation, lifestyle, WCRF/AICR, inflammatory markers

## Abstract

**Simple Summary:**

Large intestinal cancer is one of the most common lifestyle-related types of cancer. However, the exact mechanism in the relation between adipose tissue, systemic inflammation and intestinal cancer remains unknown. Furthermore, there are many hypotheses regarding the pathways in which alcohol consumption, smoking, malnutrition and physical inactivity cause low-grade systemic inflammation. In this paper, we discuss the relation of lifestyle with inflammation, in terms of inflammatory markers at the time of diagnosis in intestinal cancer patients. Our findings suggest that an overall unhealthier lifestyle and a higher risk of malnutrition at the time of diagnosis were associated with elevated levels of inflammatory markers. These findings could contribute to formulating lifestyle advice in the future.

**Abstract:**

Colorectal cancer is one of the most common lifestyle-related types of cancer. The exact pathophysiologic mechanism in the relation between (visceral) adipose tissue, systemic inflammation and colorectal cancer remains unknown. This study aimed to assess the association of lifestyle with markers of systemic inflammation at the time of diagnosis in stage I-III colorectal cancer patients. Patients (*n* = 298) with stage I-III colorectal cancer from three Dutch hospitals were included at diagnosis. Several lifestyle-related variables (MUST nutritional status score, WCRF/AICR healthy lifestyle score, active smoking, alcohol consumption and BMI) and inflammatory markers (plasma levels of IL-6, IL-8, IL-10, TNFα and ‘high sensitive’ hsCRP) were measured at the time of diagnosis. Confounder-adjusted multivariable linear regression models were used to analyse how the lifestyle variables were associated with the inflammatory markers. Statistically significant associations were found between a better WCRF/AICR lifestyle score and lower levels of IL-6 and hsCRP. A medium and high risk of malnutrition according to the MUST score was associated with elevated levels of both IL-8 and hsCRP. An overall unhealthier lifestyle indicated by a lower WCRF/AICR lifestyle score and a higher risk of malnutrition according to the MUST score at the time of diagnosis was associated with elevated levels of inflammatory markers. These findings can contribute to formulating lifestyle advice to improve treatment outcomes and prognosis in patients having CRC in the future.

## 1. Introduction

Colorectal cancer (CRC) is currently the third most common malignancy, only after breast and lung cancer, with almost 1 million new cases diagnosed worldwide annually [1]. Incidence is still rising, and there is a worrying trend of increasing incidence in young adults aged 30–39 years [2]. 

In recent years, there is an increased scientific interest in the effect of lifestyle on the development of cancer [3]. Thus far, several forms of cancer have been appointed as lifestyle-related types of cancer, i.e., endometrial carcinoma, lung cancer, pancreatic cancer, post-menopausal breast cancer and CRC [4,5]. The evidence has been predominantly based on body mass index (BMI) and body composition. The increase in risk of the development of colon cancer accumulates for every five points in BMI, starting from BMI 25 kg/m^2^ with 9% for women and 24% for men [6]. Furthermore, an increase in waist circumference of 2 cm is associated with a 4% greater risk of CRC in the general population [7]. 

The exact pathophysiologic mechanism in the relation between (visceral) adipose tissue and the occurrence of CRC is still unknown. One of the hypotheses is that the pro-inflammatory cytokines generated from the adipose tissue induce metabolic reprogramming in colon cancer cells [8]. These cytokines contribute to the systemic low-grade inflammation that is associated with obesity. It is thought that this inflammatory state further contributes to the increased risk of CRC [9,10]. 

There are also many hypotheses regarding the pathways through which alcohol consumption, smoking, malnutrition and physical inactivity may cause low-grade systemic inflammation [11,12,13,14]. Chronic alcohol consumption can lead to an increase in oxidative stress and inflammatory cell responses. Furthermore, acetaldehyde, a metabolite of alcohol, disrupts DNA repair and synthesis, contributing to the carcinogenic cascade [15,16]. Smoking is also associated with an increase in oxidative stress, which activates inflammatory response pathways [17]. Overall, lifestyle is an important factor in a chronic, low-grade systemic inflammatory state [18]. 

Physical exercise has been shown to lower circulating levels of interleukins (IL-6 and IL-8), tumour necrosis factor (TNF) α and C-reactive protein (CRP) in breast cancer survivors after treatment [19,20]. The role of inflammation has also been extensively investigated in the context of detection of post-operative complications or even local recurrence of CRC [21,22,23]. The low-grade systemic inflammatory state may also be partially maintained by the tumour itself [24]. However, little is known regarding the association between lifestyle and inflammation at the time of diagnosis in CRC patients. 

In view of these knowledge gaps, we investigated the association of lifestyle with inflammation, in terms of inflammatory markers at the time of diagnosis in CRC patients. The World Cancer Research Fund (WCRF)/American Institute for Cancer Research (AICR) has composed a core set of lifestyle recommendations in order to prevent lifestyle-related types of cancer [4]. We hypothesized that less concordance with the WCRF/AICR recommendations is associated with a higher grade of systemic inflammation, expressed in raised inflammatory markers, at the time of CRC diagnosis. The research findings could potentially provide guidance for lifestyle interventions to improve treatment outcomes and prognosis in patients with CRC.

## 2. Materials and Methods

We used cross-sectional data from an ongoing, multi-centre, prospective cohort study in the Netherlands, enrolling CRC patients since 2012. This EnCoRe study (Energy for life after ColoRectal cancer; NL6904; trialregistrer.nl) recruits stage I-III CRC patients upon diagnosis in three Dutch hospitals: the Maastricht University Medical Centre+, the VieCuri Medical Centre and the Zuyderland Medical Centre. Data collected at diagnosis were used for the present analysis. Elaborate descriptions of the design of the EnCoRe study have been provided elsewhere [25]. Briefly, patients 18 years or older with stage I-III colon or rectal cancer were included at diagnosis. Tumour characteristics were provided according to the 8th edition of the tumour–node–metastasis (TNM) classification [26]. Patients were excluded in case of stage IV disease, when unable to speak and understand the Dutch language, if their home address was not situated in The Netherlands or in case of comorbidities obstructing participation (e.g., cognitive impairment, severe visibility or hearing disorders). Data were collected at the patients’ home, at the time of diagnosis, prior to the start of (neo-adjuvant) treatment. Written informed consent was obtained from all participating patients, and ethical approval was provided by the Medical Ethics Committee of the Maastricht University Medical Centre+. All participating patients signed written consent. In total, data at time of diagnosis were available for 298 patients. 

Patients reported sex, age, level of education and use of non-steroidal anti-inflammatory drugs (NSAIDs) at the time of diagnosis. Stage of disease, tumour localisation, medical history and comorbidities were obtained from the electronic patient records.

Patients completed a validated food frequency questionnaire (FFQ) at the time of diagnosis to retrospectively assess dietary habits and alcohol consumption in the year before diagnosis [27]. Based on the FFQ, total dietary energy intake (kcal/week) was determined, as well as intake of nutrients and specific foods. The alcohol intake of patients was classified according to the guidelines of the National Institute on Alcohol Abuse and Alcoholism (NIAAA) [28,29] into occasional (0–14 g/day), moderate (14–41.9 g/day) and heavy (≥42 g/day) drinking. 

Body mass index (BMI) was calculated from the body weight and height that were measured at diagnosis. BMI was divided into the subgroups ‘healthy weight’ (18.5–24.9 kg/m^2^), ‘overweight’ (25–29.9 kg/m^2^) and ‘obese’ (≥30 kg/m^2^). 

Physical activity was self-reported by patients through the validated SQUASH questionnaire (Short QUestionnaire to ASsess Health-enhancing physical activity), assessing habitual physical activity levels in the two months before diagnosis [30]. All activities with a metabolic equivalent value ≥ 3 were used to determine hours per week of moderate-to-vigorous physical activity (MVPA) according to the compendium of physical activities [31]. Both current and past smoking behaviour were queried at diagnosis. Patients were classified as an active smoker ‘yes/no’. Questionnaires were rigorously checked on completion by the research group; patients were contacted for further clarification if needed. 

The risk of malnutrition was measured by means of the Malnutrition Universal Screening Tool (MUST) score [32]. The MUST score is composed of three independent criteria: BMI, unintentional weight loss in percentage of total body weight and the acute disease effect. The MUST score was retrieved from the electronic patient records and defined as low (0), medium (1) or high (2) risk. An overall lifestyle score was calculated based on the WCRF/AICR cancer prevention recommendations regarding body weight, physical activity and diet [4]. In 2018, the WCRF/AICR updated their lifestyle recommendations, which have been operationalized into a lifestyle scoring system [27,33,34,35]. The lifestyle score, representing seven recommendations, ranges from 0 to 7 points. Higher scores indicate adherence to more lifestyle recommendations, reflecting a healthier lifestyle. The recommendations encourage people to be a healthy weight, engage in physical activity and consume a healthy diet (eat sufficient amounts of fruits and vegetables and dietary fibre, limit intake of fast foods and red or processed meat), including limited consumption of sugary and alcoholic drinks. As previously described in detail [33], each of these recommendations is assigned 0 points if the recommendation is not met, 0.5 point if it is partially met and 1 point if the recommendation is completely met. Two of the recommendations (body weight and dietary fibre) include sub-recommendations, including the possible scores for these recommendations as 0, 0.25, 0.5, 0.75 and 1. 

The inflammatory markers IL-6, IL-8, IL-10, TNFα (pg/mL) and hsCRP (µg/mL) were determined in fasting blood samples collected during home visits at diagnosis. These inflammatory markers have been selected because of their hypothesized role in the development and progression of CRC [36]. Detailed information about the analytical methods of these samples has been published elsewhere [37]. Plasma concentrations of the inflammatory markers were log-transformed because most markers were right-skewed.

All statistical analyses were carried out using IBM SPSS Statistics (version 22: IBM corporation, Armonk, NY, USA). Descriptive analyses of clinical and socio-demographic characteristics of the participants were performed. Confounder-adjusted multivariable linear regression models were used to assess associations of several lifestyle variables (BMI, WCRF/AICR lifestyle score, MUST nutritional status score, physical activity, active smoking and alcohol intake) with the five inflammatory markers (IL-6, IL-8, IL-10, TNFα and hsCRP). Associations are presented as betas with 95% confidence intervals. In addition, correction for multiple testing using a false discovery rate (FDR) was implemented to adjust *p*-values per individual exposure for the five different models. The confounders were defined prior to the analyses and included age (years), sex (male or female), stage of disease (I-III), intake of NSAIDs at least on day of the week (aspirin and COX-2 inhibitors), total energy intake (kcal/week) and the number of comorbidities (0, 1, 2 or more). 

## 3. Results

In total, 298 patients were included in this study. The population consisted of 201 (67.4%) male and 97 (32.6%) female patients with a mean age of 66.7 (36–90) years. Baseline characteristics of the included patients and tumour characteristics are provided in Table 1. 

The mean BMI was 28.2 kg/m^2^ (SD 4.64), ranging from 18.2 to 46.0 kg/m^2^. Six patients classified as ‘morbidly obese’ with a BMI ≥ 40 kg/m^2^. Another 99 patients were ‘obese’ with a BMI ≥ 30 kg/m^2^. One patient was underweight with a BMI of 18.2 kg/m^2^. For statistical analyses, this patient was included in the healthy weight category. Furthermore, eighteen patients (6%) classified as a medium risk for malnutrition and eight patients (2.7%) were high risk for malnutrition according to the MUST score (Table 1). 

Whereas only one patient reported to not consume any alcohol, most patients were classified as occasional users (62.8%), while 32.6% and 4.4% of patients were classified as moderate or heavy alcohol users, respectively. In total, 39 patients (13.1%) were active smokers, 161 patients (54%) were former smokers and 92 patients (30.9%) had never smoked before. Patient-reported hours per week in MVPA varied widely, with an average of 15.2 h per week (SD 14.6). Patients scored 3.2 points on the WCRF/AICR score on average, with a range of 0.8–5.3. 

Concentrations of the inflammatory markers varied widely between patients (Table 2). We found slightly elevated levels of IL-8 and hsCRP in this patient cohort. For IL-6 and IL-8, TNFα and hsCRP, the median concentrations were highest in patients with stage II disease, as opposed to stage I and III. 

The results of the linear regression analysis are provided in Table 3. The analysis showed an association between a higher WCRF/AICR lifestyle score and decreased levels of IL-6 (β per 1 point: −0.13, 95% CI: −0.29, −0.02, *p* = 0.007, after FDR *p* = 0.035) and hsCRP (β per 1 point: −0.14, 95% CI: −0.44, −0.01, *p* = 0.015, after FDR *p* = 0.0375). A medium and high risk of malnutrition according to the MUST score was associated with higher levels of IL-8 (β medium and high risk vs low risk: 0.14, 95% CI: 0.25, 0.52, *p* = 0.038, no longer statistically significant after FDR with *p* = 0.095) and hsCRP (β medium and high risk vs low risk: 0.17, 95% CI: 0.14, 1.32, *p* = 0.009, after FDR *p* = 0.045). No significant associations were seen between the lifestyle variables and IL-10 or TNFα. Active smoking, alcohol consumption, BMI and hours per week spent in MVPA were not significantly associated with any of the inflammatory markers. 

## 4. Discussion

In this cross-sectional analysis among 298 patients diagnosed with stage I-III CRC, an overall unhealthier lifestyle indicated by a lower WCRF/AICR lifestyle score and a higher risk of malnutrition according to the MUST score at the time of diagnosis was associated with elevated levels of inflammatory markers. To our knowledge, this is the first study suggesting that there is an association between lifestyle factors and inflammatory markers at the time of diagnosis in patients with stage I-III CRC.

An increasing amount of evidence indicates that an increased level of systemic inflammation may affect every stage in the pathogenesis of CRC [24,38]. Inflammation may have an effect on carcinogenesis by promoting cancer growth and shifting metabolic pathways. Furthermore, systemic inflammation results in further CRC progression and metastasis and is also an independent indicator of less favourable oncological outcomes in the long-term in terms of shorter disease-free time and overall survival [39,40,41,42].

The reported increased serum levels of acute phase proteins such as CRP have previously been shown to contribute to the tumour itself and cancer-associated systemic inflammation [24,42]. We hypothesize that characteristics of an overall unhealthy lifestyle pattern also contribute to systemic inflammation and thus would be associated with elevated levels of inflammatory markers at diagnosis.

An extensive amount of research has already demonstrated that CRC is one of many lifestyle-related types of cancer [3,4,5]. The presence of a surplus of visceral fat is thought to enhance a chronic inflammatory response. Furthermore, high levels of insulin can lead to cell growth and inhibit apoptosis [43,44]. Naturally, lifestyle is a far broader concept than weight or BMI alone. For instance, the consumption of both processed and red meats has been known to modestly but significantly increase the risk of CRC [45,46].

Alcohol is known to have a genotoxic and carcinogenic effect because of increased oxidative stress and thus the production of reactive oxygen species [47,48]. Active smoking has been demonstrated to increase both incidence of and mortality in CRC [49]. Yang et al. [50] found an association between a history of smoking and left-sided colon cancer, and this relation was even stronger for rectal cancer in patients with more than 30 pack-years. Several studies have already shown that inflammatory markers, especially CRP, IL-6 and leukocytes, are raised in active smokers [12,51,52]. Our findings did not reach statistical significance to support this association, possibly due to a limited number of active smokers (N = 39) within our population.

A meta-analysis by Bano et al. [53] has shown that CRP levels are likely to be elevated in sarcopenic CRC patients. This supports our findings of statistically significant elevated hsCRP levels in patients with higher MUST scores.

The anti-inflammatory effects of physical exercise may be mediated via multiple pathways. Both a reduction in visceral fat as a result from a change in body composition and the creation of an ‘anti-inflammatory environment’ may contribute [13,54]. No association was observed between MVPA and increased levels of the inflammatory markers in our patient cohort.

Strengths of this study include the use of a comprehensive set of lifestyle recommendations and parameters for our analyses. However, potential limitations should also be considered. The fact that we have performed cross-sectional analyses prevents us from drawing conclusions on the causal direction of the observed associations. Since several of the lifestyle variables were self-reported by the patients, this may have resulted in measurement bias. Furthermore, since it is a selected patient population of only stage I-III CRC, we are not able to draw any conclusions on the implications for stage IV CRC. Previous studies have shown that systemic inflammation reactions are predominantly present in the poorly differentiated and advanced types of CRC [55,56]. Furthermore, future studies should evaluate the inflammatory markers throughout the follow-up and relate these to oncological outcomes.

We are optimistic that these preliminary findings of the association between an unhealthier lifestyle and elevated inflammatory markers can be a starting point for concrete lifestyle advice in the future, in order to improve treatment outcomes and prognosis in patients with CRC. More research is needed in order to fully comprehend the association between lifestyle and inflammatory markers before the diagnosis of CRC.

## 5. Conclusions

Several lifestyle factors were shown to be significantly associated with markers of elevated inflammation at the time of CRC diagnosis. An overall unhealthier lifestyle indicated by a lower WCRF/AICR lifestyle score and a higher risk of malnutrition according to the MUST score at the time of diagnosis was associated with elevated levels of inflammatory markers. These findings might provide leads for improving lifestyle advice for newly diagnosed CRC patients in the future.

## Figures and Tables

**Table 1:** Sociodemographic and lifestyle characteristics of the study population (N = 298).

Patient Characteristics	Mean (SD) or Number (%) as Indicated
Age, mean (SD ^1^)	66.7 (9.3)
Sex, N (%)	
Male	201 (67.4%)
Female	97 (32.6%)
Tumour localization, N (%)	
Right colon	65 (21.8%)
Transverse colon	10 (3.4%)
Left colon	18 (6.0%)
Sigmoid	87 (29.2%)
Rectum	118 (39.6%)
Stage of disease, N (%)	
I	90 (30.2%)
II	67 (22.5%)
III	141 (47.3%)
BMI ^2^	28.24 (4.8)
Healthy weight ^3^ (18-<25)	80 (26.8%)
Overweight (25-<30)	119 (39.9%)
Obese (≥30)	99 (33.2%)
WCRF/AICR score ^4^ (0–7), mean (SD)	3.2 (0.8)
MUST score ^5^ (0–2), N (%)	
Low risk (0)	236 (79.2%)
Medium risk (1)	18 (6.0%)
High risk (2)	8 (2.7%)
Unknown	36 (12.1%)
MVPA ^6^ (h/wk), mean (SD)	15.15 (14.6)
Smoking status ^7,^ N (%)	
Yes	39 (13.1%)
No	253 (84.9%)
Alcohol intake (g/day), Mean (SD)	12.5 (15.1)
Occasional (0–14 g/day)	187 (62.8%)
Moderate (14–42 g/day)	97 (32.6%)
Heavy (>42 g/day)	13 (4.4%)
Cox-2 inhibitor use (weekly), N (%)	14 (4.7%)
Comorbidities	
No	59 (19.8%)
Yes,1	65 (21.8)
Yes, 2 or more	174 (58.4%)

^1^ SD: standard deviation, ^2^ BMI: body mass index, ^3^ One ‘underweight’ patient with a BMI of 18.2 kg/m^2^ was included in this subgroup, ^4^ WCRF/AICR: World Cancer Research Fund/ American Institute for Cancer Research, ^5^ MUST: Malnutrition Universal Screening Tool. Missing data in 36 patients (12.1%), ^6^ MVPA: moderate-to-vigorous physical activity, ^7^ Missing data in six patients (2%).

**Table 2:** Inflammatory markers at diagnosis (N = 298).

Subgroups	IL-6 ^3^ (pg/mL)	IL-8 (pg/mL)	IL-10 (pg/mL)	TNF-α ^4^ (pg/mL)	hsCRP ^5^ (μg/mL)
**Concentrations**					
Median (IQR ^1^)	1.07 (0.98)	5.68 (3.91)	0.25 (0.21)	2.21 (0.97)	2.89 (5.89)
**Male**	1.04 (1.06)	5.50 (4.25)	0.25 (0.21)	2.23 (1.02)	2.45 (5.51)
**Female**	1.18 (0.95)	6.09 (4.93)	0.23 (0.17)	2.18 (1.18)	3.49 (5.88)
**Stage of disease**					
** I**	1.12 (1.04)	5.39 (5.33)	0.23 (0.17)	2.18 (0.81)	2.96 (7.26)
** II**	1.79 (0.83)	7.01 (4.43)	0.22 (0.25)	2.27 (1.02)	2.89 (5.70)
** III**	1.03 (1.23)	5.90 (4.13)	0.27 (0.21)	2.24 (1.30)	2.73 (5.74)
**No weekly NSAID ^2^ use**	1.07 (0.96)	5.77 (4.33)	0.24 (0.20)	2.20 (1.06)	2.89 (5.60)
**Weekly NSAID use**	1.16 (1.08)	5.64 (3.83)	0.30 (0.17)	2.61 (1.07)	2.87 (5.38)

^1^ IQR: interquartile range, ^2^ NSAID: non-steroidal anti-inflammatory drug, ^3^ IL: interleukin, ^4^ TNFα: tumour necrosis factor alpha, ^5^ hsCRP: high sensitive C-reactive protein.

**Table 3:** Association between lifestyle factors and inflammatory markers in stage I-III colorectal cancer patients at diagnosis—a multivariable linear regression analysis.

	IL-6 ^7^		IL-8 ^7^		IL-10 ^7^		TNF-α ^7^		hsCRP ^8^	
	β	95% CI	β	95% CI	β	95% C	β	95% CI	β	95% CI
**BMI ^1^** **25.0–29.9**	0.07	−0.14–0.38	−0.04	−0.21–0.12	−0.01	−0.26–0.23	0.08	−0.04–0.16	−0.02	−0.45–0.36
**BMI ≥ 30**	0.13	−0.04–0.53	−0.07	−0.26–0.10	0.04	−0.20–0.34	0.08	−0.05–0.17	0.01	−0.43–0.45
**MUST ^2^**	0.07	−0.16–0.52	**0.14**	**0.25–0.52**	−0.01	−0.37–0.35	0.04	−0.09–0.19	**0.17**	0.14–1.32 *
**WCRF/AICR ^3^**	**−0.13**	−0.29–0.02 *	−0.01	−0.09–0.09	−0.03	−0.17–0.10	−0.11	−0.10–0.01	**−0.14**	−0.44–0.01 *
**Smoking ^4^**	0.08	−0.09–0.52	0.07	−0.85–0.31	0.02	−0.25–0.33	0.09	−0.02–0.21	0.12	−0.04–0.89
**Alcohol ^5^**	−0.11	−0.42–0.01	−0.01	−0.15–0.13	0.06	−0.11–0.30	−0.06	−0.13–0.04	−0.06	−0.49–0.20
**MVPA ^6^**	−0.03	−0.01–0.01	0.03	−0.01–0.01	−0.01	−0.01–0.01	−0.03	−0.01–0.01	0.04	−0.01–0.02

^1^ BMI in kg/m^2^, with healthy weight (18.5-24.9) as reference category. ^2^ MUST: Malnutrition Universal Screening Tool. Medium and high risk versus low risk. ^3^ World Cancer Research Fund/ American Institute for Cancer Research score, score ranging from 0-7. ^4^ Active smokers versus former smokers and patients without a history of smoking. ^5^ Alcohol intake: moderate and heavy drinkers versus occasional drinkers. ^6^ MVPA: moderate-to-vigorous physical activity in hours/week. ^7^ The values of the inflammatory markers IL-6, IL-8, IL-10, and TNFα are the log2-transformed values of the plasma concentrations (original concentration in pg/mL). ^8^ The values of the inflammatory marker hsCRP are the log2-transformed values of the plasma concentrations (original concentration in μg/mL). All analyses are corrected for: sex, age, stage of disease, number of comorbidities, total energy intake per week in kcal and the weekly use of NSAID by including the confounders as predictors in the multivariable regression model. All values in bold print were statistically significant; *p* < 0.05. * Indicates the *p*-values that remained statistically significant after false discovery rate (FDR) correction for multiple testing.

## Data Availability

The data presented in this study are available on request from the contact person for the EnCoRe study (M.J.L Bours). The data are not publicly available due to privacy restrictions.
